# Does dispositional envy predict depression severity in Ukrainian adults living in wartime?

**DOI:** 10.3389/fpsyg.2026.1774123

**Published:** 2026-03-25

**Authors:** Mariana Velykodna, Valeriia Popudrenko, Viktoriia Yeromina, Elizaveta Musiyaka, Nataliia Chernysh, Vladyslav Deputatov

**Affiliations:** 1 Psychology Department, Ukraine Sigmund Freud University, Kyiv, Ukraine; 2 Practical Psychology Department, Kryvyi Rih State Pedagogical University, Kryvyi Rih, Ukraine

**Keywords:** depression, dispositional envy, envy, the 2022 Russian invasion, Ukraine, war

## Abstract

**Objective:**

Russia’s war against Ukraine, which escalated in 2022, with related losses, injuries, and forced displacements, resulted in numerous mental health issues in Ukrainians, including a rise in the prevalence of depression. Researchers report various psychosocial factors that predict, mediate, or moderate depressive symptoms in Ukrainian adults facing war. However, among psychological factors, they focus mostly on resilience or wellbeing levels. In contrast, personality traits, including dispositional envy, were not explored enough.

**Methods:**

This study was organized as an online survey, which included a questionnaire on sociodemographic data, Dispositional Benign and Malicious Envy Scale (BeMaS), and PHQ-9 for depression assessment. 3,026 Ukrainian adults (52.2% males; 76.5% married) aged from 18 to 74 (M = 42.4, SD = 9.72) filled in the survey in January–March 2025. The TRIPOD checklist for prediction model development and validation was utilized for the transparency of this report.

**Results:**

Although dispositional malicious envy weakly correlated with depressive symptoms (*r* = 0.039, *p* < 0.05), both dispositional envy types did not succeed in explaining depression levels more than 0.2% of its variance. Female gender, younger age, and higher educational degrees, along with malicious envy, contributed to depression altogether, explaining only 4.6% of its variance. Interestingly, both dispositions of envy correlated with each other (*r* = 0.381, *p* < 0.001).

**Conclusion:**

Dispositional envy type does not predict depression levels in Ukrainian adults during wartime. Generally, the role of envy in the development of depressive symptoms in crisis circumstances may be overrated.

## 1 Introduction

The ongoing Russia’s war against Ukraine, which started in 2014 and increased in scale in 2022, is considered the largest artificial catastrophe in Europe since World War II (Delanty, 2023). Researchers reported a dramatic growth of the prevalence and severity of mental health issues within Ukraine (Serdiuk et al., 2025) and abroad (Esbit et al., 2025) in response to this war, including a reflected increase of psychiatric hospitalizations (Barlattani et al., 2023; Pinchuk et al., 2025).

Depressive symptoms are often reported among other mental health sufferings prevalent in the war-exposed population of Ukrainians (Barlattani et al., 2023). By October 2023, 33.6% of university students showed moderate or severe levels of depression measured by PHQ-9 (Polyvianaia et al., 2025). By December 2024, moderate to severe depression was prevalent among 61.79% of Ukrainian women and 65.96% of men (Kurapov et al., 2025). In men, it also resulted in more frequent suicidal thoughts, while women reported a feeling of helplessness more often (An et al., 2025). Along with seeking appropriate medication for clinical depression developed in wartime (Sikora et al., 2023), current research efforts are focused on revealing at-risk groups and providing relevant and effective interventions for specific war-associated symptoms, which are required both in clinical and public health settings (Fornaro et al., 2025).

Recent studies report various psychosocial factors that predict, mediate, or moderate depressive symptoms in Ukrainian adults facing war. Female gender, older age, direct war trauma exposure, and fleeing to other countries (Kurapov et al., 2023), significant losses (Urbański et al., 2024), having children (Fel et al., 2023), financial issues, and lack of social support (Timmer et al., 2023) were associated with more severe depression levels. However, among psychological factors, prior studies focus mostly on resilience (Humphrey and Forbes-Mewett, 2025; Urbański et al., 2024), self-efficacy (Kokun, 2024), or wellbeing levels (Pinchuk et al., 2024a, 2024b). In contrast, personality traits, including dispositional envy, were not explored enough.

Dispositional envy is a stable personality trait that reflects a tendency towards benign or malicious responses (Erz and Rentzsch, 2024) in one’s episodic experience of envy (Cohen-Charash, 2009), characterized by an increase in inspiration and motivation towards achievements versus hostility and self-blaming (Lange et al., 2017; Ng et al., 2021). Following previous studies, dispositional envy significantly contributes to life satisfaction, happiness, and vitality, including by increasing the feeling of low energy (Milfont and Gouveia, 2009). Although research suggests that relationships between dispositional envy and depression symptoms are unidirectionally causal (Jiang and Wang, 2020), they are intensively discussed as dynamic and complex (Mehrabi et al., 2024), and moderated by social variables (Xiang et al., 2020), particularly social comparison (Li, 2019). As malicious envy promotes and benign envy inhibits the effects of maltreatment on depression (Li et al., 2022), a similar role of dispositional envy type could be expected in the severity of depressive symptoms during hostilities of military aggression. However, there is a lack of data on the contribution of envy in developing depression in crisis circumstances such as war.

This paper presents the results of the prediction model development and validation, answering the question, «Does Dispositional Envy Predict Depression Severity in Ukrainian Adults Living in Wartime?»

## 2 Materials and methods

The research design followed the TRIPOD+AI Checklist for Prediction Model Development (Collins et al., 2024). The study was organized as a cross-sectional and prediction model development aimed at revealing associations, in which one of the tested variables predicts the size of another one, considered an outcome, at this very moment. However, this approach does not allow exploring the anticipation of developing the tested outcome (depression) as a consequence of the tested variables (dispositional envy types) on a timeline, as it could be investigated through a longitudinal study.

### 2.1 Outcome

Depression levels measured using the Patient Health Questionnaire PHQ-9 (Kroenke et al., 2001), recently reassessed for the Ukrainian population (Kohut and Chaban, 2025), were chosen as an outcome in this study. PHQ-9 is a 9-item 4-point Likert scale. When using in public screening, PHQ-9 scores above 10 are considered a threshold for possible clinical depression. Cronbach’s alpha calculation supported the reliability of the one-factor structure of this scale in our sample (0.876).

### 2.2 Tested variables

The list of the tested variables utilized in the prediction model development included: sociodemographic nominal variables (e.g., age, sex, marital and working status, and education levels) and dispositional envy types, measured via the Dispositional Benign and Malicious Envy Scale (BeMaS; Lange and Crusius, 2024), adapted for the Ukrainian population (Pylat and Haletska, 2022). BeMaS is a 10-item 6-point Likert scale with Benign Envy and Malicious Envy as its subscales. Cronbach’s alpha calculation supported the reliability of the two-factor structure of this scale in our sample (0.831 and 0.923).

### 2.3 Participants

We aimed to recruit a representative sample of adult (above 18 years old) Ukrainians throughout the country. An online survey was chosen as the most appropriate instrument for this purpose. Considering the recent studies reporting mental health conditions in Ukraine, with the majority of female respondents (e.g., Polyvianaia et al., 2025), we attempted to involve equal gender groups of participants. Expected sample size was estimated as a minimum of 1849 full responses, with reflected 99% confidence level with 3% margin of error for the general population of Ukrainians.

### 2.4 Data collection

The survey, constructed via Google Forms, was distributed from January to March 2025 through several channels, including social media and large corporations’ workers’ email lists. It consisted of the research description, written consent for participation, and a questionnaire with measured variables.

### 2.5 Data curation

The research plan included excluding the responses with missing data of the outcome (PHQ-9) and key variables (Benign and Malicious Envy scales). However, missing data were not observed for these variables; therefore, no imputation was applied. Multicollinearity was assessed using variance inflation factors (VIFs), indicating negligible collinearity (VIFs = 1.01–1.03; tolerances = 0.97–0.99). Regression assumptions were examined using residual plots and Q–Q plots of residuals. Given departures from normality of residuals typical for symptom severity scores, we conducted a sensitivity analysis using a negative binomial generalized linear model (GLM) for the PHQ-9 total score. In interpreting results, we emphasize effect sizes (standardized coefficients and explained variance, *R*^2^) and their confidence intervals, as statistical significance can be inflated in very large samples; we do not apply rigid cut-offs when characterizing magnitude.

### 2.6 Data analysis

The data analysis was performed using the R-based software Jamovi 2.3.28, and included descriptive statistics, Cronbach’s alpha calculation for the scales’ reliability assessment, and linear regression analysis for developing a multivariable prediction model. The linear regression analyses included both unadjusted and covariate-adjusted models, and the corresponding model fit indices (*R*^2^ and adjusted *R*^2^) were reported in the regression tables.

### 2.7 Ethical approval

The ethical approval for this study was obtained from the Ethical Board of the Ukraine Sigmund Freud University.

## 3 Results

### 3.1 Participants

3,026 Ukrainian adults aged 18–74 years old (M = 42.4, SD = 9.72, Mode = 42, SE = 0.18) provided full responses to the survey. 52.2% were males, 98.9% were employed, and 76.5% married. Most had a bachelor’s or master’s diploma (63.1%), while 35.3% had a professional graduate diploma (i.e., graduated from a college), 1.3% graduated from school, and 0.3% had a doctoral degree. Table 1 shows the means and standard deviation of participants’ dispositional envy and depression levels. 24.5% of participants reached a cut-off of 10 points in depression levels hypothesized as possible markers of clinical depression.

**Table 1:** Descriptives of scales.

Variable	M	Me	SD	Min–max
Benign envy	13.1	13	4.05	5–20
Malicious envy	8.21	10	2.95	5–20
Depression	6.55	5	5.61	0–27

### 3.2 Prediction model development

The prediction models for depression scores as an outcome in this study were developed as follows. First, we used two separate linear regression analyses to assess the performance of two dispositional envy types as independent variables in predicting the depression levels. After this procedure, due to the poor role of the tested variables in explaining depression (see section 3.3), we developed a multivariable predictive model, using two dispositional envy types and evidence-driven sociodemographic parameters as independent variables for depression severity. In developing this third model, we were mainly focusing on the means of *R*^2^. As the purpose of its development was exploratory search for other potential predictors among the collected data, each tested variable was added to the model one by one and left in it or rejected depending on its *p*-value (*p* < 0.05 was chosen as a cutoff for this process). Changes in the *p*-value and *β*-value of the rest of the variables, as well as the success of the developed model in general, were also considered.

### 3.3 Prediction models performance

Following the described procedure, we developed several prediction models with unsatisfactory properties, which are presented in Tables 2, 3. The first model evaluated the performance of Benign dispositional envy in predicting depression, and the second assessed the predictive value of Malicious envy. The third model included Dispositional envy scales and social variables.

**Table 2:** Linear regression models predicting depression.

Model	*R*	*R* ^2^	Adjusted *R*^2^	Overall model test			
				*F*	df1	df2	*p*
1	0.008	0.00007	−0.0002	0.199	1	3,024	0.656
2	0.039	0.00152	0.00119	4.61	1	3,024	0.032
3	0.215	0.0461	0.0448	36.5	4	3,021	<0.001

**Table 3:** Predictors of depression.

Effect	Estimate	SE	95% CI		*p*	Stand. estimate (LL: UL)
			LL	UL		
Model 1						
Intercept	6.4	0.35	5.73	7.08	<0.001	
Benign envy	0.01	0.03	−0.02	0.06	0.656	0.01 (−0.03: 0.04)
Model 2						
Intercept	5.94	0.30	5.35	6.53	<0.001	
Malicious envy	0.07	0.03	0.001	0.142	0.032	0.04 (0.00: 0.08)
Model 3						
Intercept	9.38	0.53	8.34	10.41	<0.001	
Gender^a^	1.6	0.2	1.2	2	<0.001	
Professional graduate diploma^b^	−1.23	0.21	−1.64	−0.81	<0.001	
Malicious Envy	0.13	0.03	0.065	0.199	<0.001	0.07 (0.03: 0.11)
Age	−0.06	0.01	−0.082	−0.041	<0.001	−0.11 (−0.14: −0.07)

a Gender coded 0 = male, 1 = female (reference category = male).

b Education (highest level) was dummy-coded. 1 = “Professional graduate diploma” if this was the highest educational level, 0 = otherwise (reference category = all other education levels).

CI = confidence interval; LL = lower limit; UL = upper limit. Standardized estimates are reported for continuous predictors; for categorical predictors, unstandardized coefficients are shown with the reference category indicated.

The first model was not successful in explaining the variance of depression symptoms severity (*R*^2^ = 0.007; *F* = 0.199, *p* = 0.656) with Benign envy (*β* = 0.0113, *p* = 0.656). The second model succeeded to explain 0.15% (*F* = 4.61, *p* < 0.032) of depression levels’ variance with Malicious envy (*β* = 0.074, *p* = 0.032).

The third, most successful, model explained only 4.6% (*F* = 36.5, *p* < 0.001) of depression variance with 4 predictors. The variables that predicted higher depression levels were female gender (*β* = −1.6, *p* < 0.001), and dispositional malicious envy (*β* = 0.132, *p* < 0.001). Instead, predictors for the lower depression levels were professional graduate degree (*β* = −1.225, *p* < 0.001), and older age (*β* = −0.062, *p* < 0.001).

As a sensitivity analysis, a negative binomial GLM yielded results consistent with the OLS models. Malicious envy remained positively associated with PHQ-9 but with a very small effect (IRR = 1.019, 95% CI [1.008, 1.030]). We tested interactions between malicious envy and socio-demographic variables in sensitivity models. The malicious envy × gender and malicious envy × education interactions were not significant (*p* > 0.05). The malicious envy × age interaction reached statistical significance, but the moderation effect was extremely small (B = −0.00131; IRR ≈ 0.999), indicating a marginal attenuation of the envy–PHQ-9 association with increasing age.

### 3.4 Other findings

Both dispositions of envy had a moderate correlation with each other (*r* = 0.381, *p* < 0.001), and malicious envy weakly correlated with depressive symptoms (*r* = 0.039, *p* < 0.05). The full correlation matrix is shown in Table 4.

**Table 4:** Correlation matrix of scales.

Variable	Benign envy	Malicious envy	Depression
Benign envy	1		
Malicious envy	0.381***	1	
Depression	0.008	0.039*	1

*p < 0.05, ***p < 0.001.

## 4 Discussion

In this study, we aimed to investigate whether the role of dispositional envy in the development of depression, as shown in previous research (Jiang and Wang, 2020; Mehrabi et al., 2024), is relevant to the Ukrainian population living in wartime. For this purpose, we utilized two dispositional envy types, Benign and Malicious, as hypothesized predictors of depression severity in Ukrainian adults. Sociodemographic specifics such as age, gender, educational level, working and marital status were additional variables considered in the prediction model development. As a result, we developed, tested and compared three prediction models: two for benign and malicious envy dispositions as separate variables, and one general model for envy and sociodemographic factors altogether. Generally, all three models were considered unsuccessful in predicting depression. The most successful model explained only 4.6% of the variance of depression with 4 parameters: gender, education level, malicious envy, and age.

First and foremost, both types of dispositional envy showed their poor predictive value for depression levels among adults living in wartime Ukraine. Only malicious envy disposition weakly correlated with depressive symptoms, and the final prediction model included its scores as positively associated with depression to an unsignificant extend. Overall, these results may contribute to the evidence that war-related mental health issues have their own specifics and dynamics (Khrystenko, 2026; Pinchuk et al., 2024a, 2024b), in which war exposure (Carpiniello, 2023), trauma (Velykodna et al., 2024a), and social support (Velykodna et al., 2024b) are considered more influential contributors to mental health conditions than psychological features, including personality traits such as dispositional envy.

Consistent with previous research (Bartova et al., 2021; Raccanello et al., 2024; Shi et al., 2021), including in the Ukrainian population exposed to war (Kurapov et al., 2025; Yasenok et al., 2025), female respondents self-reported higher depression levels in this study. Although it is typically associated with hormonal differences (Mengelkoch and Slavich, 2024; Wang et al., 2024), the expectation that men should be less emotionally affected should also be considered (Shi et al., 2021). While Ukrainian women tend to talk about their feelings of helplessness in response to war more openly, men tend to report having thoughts, including suicidal ones, more than they share their emotional experiences (An et al., 2025).

Higher education levels are usually associated with decreased depression levels, as education normally leads to a better quality of life in developed countries (Li et al., 2022). Moreover, in wartime Ukraine, the continuation of university education during the first year of the 2022 Russian invasion of Ukraine predicted lower depression scores in adults (Fel et al., 2023). However, this study, which utilized data collected in 2025, showed that having a professional graduate diploma instead of a bachelor’s, master’s, or doctoral degree (i.e., having a lower education level) is associated with milder depression symptoms.

Our findings regarding age as a negative predictor for depression levels contradict other studies conducted in wartime Ukraine (Fel et al., 2023; Kurapov et al., 2025) and require further investigation.

## 5 Conclusion

Overall, the current research demonstrates that dispositional envy types have a poor (almost none) predictive value for depression levels in Ukrainian adults during wartime. Generally, the role of envy in the development of depressive symptoms in crisis circumstances may be overrated compared to other war-related contributors and the quality of social support, instead of traits. Nevertheless, further investigation would benefit from exploring the role of other personality traits in depression severity and investigating the mechanisms through which envy, particularly malicious envy, might interact with war-related stressors, trauma, or social comparison processes.

## 6 Limitations

This study has several limitations. First, although the sample was large and well-representative with respect to age and gender, it was non-probabilistic, e.g., as it consisted primarily of highly educated working adults, which could influence the results. Therefore, the possibility to generalize the obtained results to the general population of Ukrainians is limited. Instead, a large sample size could influence the findings by overrating the statistical significance of the results. Second, the sociodemographic section of the questionnaire did not include information on participants’ current location or war-forced displacement of the study participants, which are known to strongly affect Ukrainians’ mental health (Barlattani et al., 2023; Ievgrashyna, 2024; Komarynska-Diament, 2025; Lushchak et al., 2024). Third, the sample included a general, i.e., non-clinical, population. Further research should focus on assessing dispositional envy among Ukrainian adults with diagnosed depressive disorder. Fourth, recent research revealed that PHQ-9 has issues with response process validity, as respondents often misunderstand the instructions of this scale (Panayiotou et al., 2025). Finally, the methodology of a predictive framework applying to the cross-sectional design allows for stating the association of measured variables, but does not explain the studied phenomena in terms of a causal relationship. The last requires a longitudinal study (Stratta et al., 2023).

## Data Availability

The datasets presented in this study can be found in online repositories. The names of the repository/repositories and accession number(s) can be found at: https://osf.io/435uh.
